# Potential use of fatty acid profiles of the adductor muscle of cockles (*Cerastoderma edule*) for traceability of collection site

**DOI:** 10.1038/srep11125

**Published:** 2015-06-18

**Authors:** Fernando Ricardo, Tânia Pimentel, Ana S. P. Moreira, Felisa Rey, Manuel A. Coimbra, M. Rosário Domingues, Pedro Domingues, Miguel Costa Leal, Ricardo Calado

**Affiliations:** 1 Departamento de Biologia & CESAM, Universidade de Aveiro, Campus Universitário de Santiago, 3810-193 Aveiro, Portugal; 2 QOPNA, Department of Chemistry, University of Aveiro, Campus Santiago, 3810-193 Aveiro, Portugal

## Abstract

Geographic traceability of seafood is key for controlling its quality and safeguarding consumers’ interest. The present study assessed if the fatty acid (FA) profile of the adductor muscle (AM) of fresh cockles (*Cerastoderma edule*) can be used to discriminate the origin of specimens collected in different bivalve capture/production areas legally defined within a coastal lagoon. Results suggest that this biochemical approach holds the potential to trace sampling locations with a spatial resolution <10 Km, even for areas with identical classification for bivalve production. Cockles further away from the inlet, i.e. in areas exposed to a higher saline variation, exhibited lower levels of saturated fatty acids, which are key for stabilizing the bilayer structure of cell membranes, and a higher percentage of polyunsaturated fatty acids, which enhance bilayer fluidity. Results suggest that the structural nature of the lipids present in the AM provides a stable fatty acid signature and holds potential for tracing the origin of bivalves to their capture/production areas.

Bivalves display a complex hydromechanical ability to filter a range of suspended particles from surrounding water^1^. Being filter feeders^2^, these molluscs are recognized for their potential to accumulate pathogenic microorganisms, thus representing a potential risk to human health when consumed raw or lightly cooked^3^. As bivalve shellfish plays an important role in global fisheries and aquaculture^4^, it is expected that supply chains commercializing live bivalves display a growing awareness towards food safety issues in an age of global trade^5^.

The microbiological safety of bivalves destined for human consumption in member states of the European Union (EU) is covered by Council Regulation 853/2004 and 854/2004^6^,^7^. Briefly, capture/production areas for bivalves in the EU are ranked according to the levels of *Escherichia coli* present in the flesh and intra-valvular liquid of live specimens and quantified through a 5-tubes 3-dilution most probable number (MPN) test. Bivalves originating from an area classified as “A” display less than 230 MPN of *E. coli* per 100 g of flesh and intra-valvular liquid. Consequently, these bivalves do not require any post-harvest treatment to reduce microbiological contamination. Bivalves originating from an area classified as “B” must not exceed in 90% of sampled specimens with 4600 MPN *E. coli* per 100 g of flesh and intra-valvular liquid, with the remaining 10% of specimens not exceeding 46000 MPN *E. coli* per 100 g of flesh and intra-valvular liquid. As a result, these bivalves must be depurated, relayed or cooked by an approved method. Bivalves originating from an area classified as “C” must not exceed the limits of MPN test of 46.000 *E. coli* per 100 g of flesh and intra-valvular liquid. These bivalves must be relayed or cooked by an approved method^6^,^7^,^8^,^9^. Different classifications may be recorded within the same estuary/coastal lagoon.

To protect public health and address current European legislation, it is paramount to trace the origin of captured/produced bivalves, not solely to a given estuary/coastal lagoon but specifically to its capture/production area. Council Regulation EC No 104/2000^10^ specifies that seafood may not be retailed unless it clearly indicates the commercial name of the species, the production method and the capture/production area. However, the specific capture/production area is usually unavailable to the end consumer. Regardless of the detail provided on the origin of traded bivalves, competent authorities should be able to trace the origin of traded specimens, thus avoiding fraud and minimizing risks to public health^11^. While most techniques employed to monitor seafood traceability are focused on issues related to species mislabeling^12^, they fail to provide detailed information on the geographic origin of seafood^13^. Nonetheless, molecular markers (e.g. microsatellites) have already been successfully used to identify the origin of bivalves (e.g. razor clam, *Ensis siliqua*) collected in locations tens to hundreds of Km apart^14^,^15^. However, this approach is unlikely to have enough resolution to discriminate specimens originating from adjacent capturing/production areas within the same estuary/coastal lagoon due to the genetic flow that occurs between these habitats. The fatty acid (FA) profile displayed by the adductor muscle (AM) of bivalves (e.g. great scallop, *Pecten maximus* and *Astarte sulcata*) has also been used to evidence significant differences in specimens originating from geographically close populations^16^,^17^. Nonetheless, it remains unclear whether this biochemical approach has enough resolution to discriminate specimens originating from adjacent areas (<10 Km apart) within the same estuary/coastal lagoon.

Ria de Aveiro is a coastal lagoon located in the western Atlantic margin of Portugal (Fig. 1a) where bivalve fisheries/aquaculture play an important socio-economic role^18^, especially the harvesting of cockle (*Cerastoderma edule*) from Ria de Aveiro, which exceeds 1000 tons per year. This coastal lagoon currently has four official bivalve capture/production areas classified either as B or C (see definition above). To provide a potential tool for bivalves traceability, the present study aimed to evaluate if the FA profile of the AM of fresh cockles could be used as a method to discriminate the origin of specimens collected in different channels of Ria de Aveiro, either with identical or different classifications for bivalve capture/production. The following null hypothesis was tested: the FA profile of the AM of *C. edule* does not differ between specimens captured in different channels of Ria de Aveiro.

## Materials and methods

### Study area and sample collection

**Figure 1:**
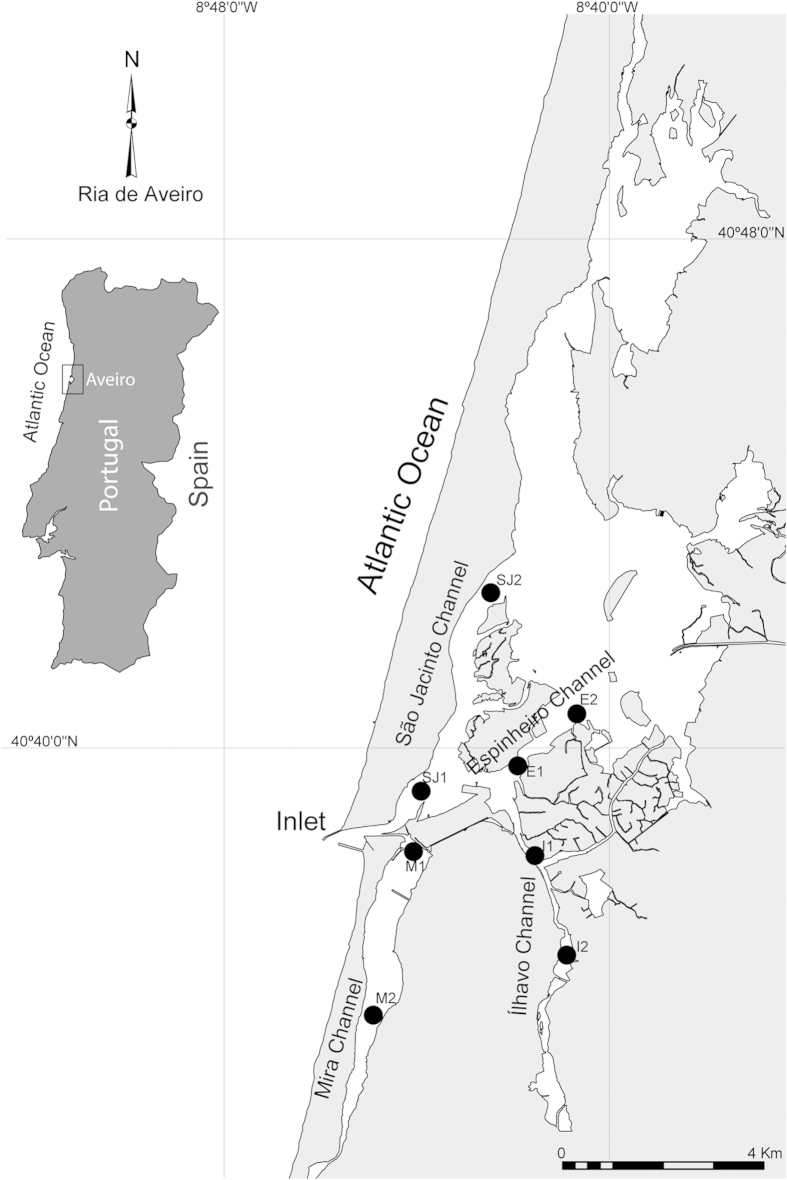
Sampling locations of *Cerastoderma edule* in Ria de Aveiro, Portugal: São Jacinto (SJ1: 40°39′23.70″N, 8°43′49.40″W and SJ2: 40°42′34.00″N, 8°42′24.10″W), Mira (M1: 40°38′26.30″N, 8°43′58.90″W and M2: 40°35′58.30″N, 8°44′47.80″W), Ilhavo (I1: 40°38′22.36″N, 8°41′24.93″W and I2: 40°37′03.10″N, 8°40′48.00″W) and Espinheiro (E1: 40°39′48.50″N, 8°41′45.03″W and E2: 40°40′37.10″N, 8°40′28.90″W). The map was created using the software ArcGIS v9.2.

Samples of *Cerastoderma edule* were collected in important fishing areas during June 2013 within four main channels (São Jacinto, Mira, Ilhavo and Espinheiro; Fig. 1) of Ria de Aveiro (Northwestern coast of Portugal), with their current classification under the legislation for shellfish production waters being used as rationale for the experimental design used (see Introduction). During the study period, the channels of São Jacinto and Mira were classified by Portuguese authorities as “B”, while those of Ilhavo and Espinheiro were classified as “C”^19^. Two areas were surveyed in each channel (Fig. 1; Table S1 on supplementary information), and four cockles collected per area (4 channels X 2 areas X 4 replicates = 32 samples). All samples were collected by hand-raking and stored in aseptic food grade plastic bags that were kept refrigerated during sampling and transportation for processing in the laboratory on the same day. Bivalves were dissected to extract the AM, which was then stored at −80 °C for subsequent FA analysis.

### Fatty acid analysis

All samples were freeze dried prior to biochemical analysis. Total lipids of the AM of each individual were extracted in methanol/chloroform (2:1 v/v) following Bligh and Dyer^20^. Fatty acids methyl esters (FAMEs) of the total lipid extracts were obtained by transmethylation according to the method described by Aued-Pimentel *et al.*^21^. Briefly, 15 μg of dried lipid extract was dissolved in 1 mL *n-hexane*, 0.2 mL of methanolic solution KOH (2 M) and 2 mL satured NaCl solution, followed by intense vortexing. After centrifugation at 2000 rpm for 5 min, the organic phase was collected and dried under a nitrogen stream. The resulting FAMEs were dissolved in hexane prior to injection and analysed by gas chromatography-mass spectrometry (GC-MS) on an Agilent Technologies 6890N Network (Santa Clara. CA) equipped with a DB-1 column with 30 m length, 0.25 mm internal diameter and 0.1 μm film thickness (J&W Scientific, Folsom, CA). The GC-MS was connected to an Agilent 5973 Network Mass Selective Detector operating with an electron impact mode at 70 eV and scanning the range m/z 40-500 in a 1 s cycle in a full scan mode acquisition. The column temperature was programmed from 40 °C initial oven temperature at 20 °C/min to 220 °C, then from 220 to 240 °C at 2 °C/min. and then from 240 to 260 °C at 5 °C/min. The detector was set at 230 °C and the injector at 220 °C. Helium was used as carrier gas at a flow rate of 1.7 mL/min. Individual FA peaks were integrated using the equipment’s software, and identified considering the retention time and mass spectrum of each FA relative to 34 mixed FA standards (C6-C24, Supelco 37 Component Fame Mix). The areas of the 20 selected FAMEs were integrated setting response factor to 1. Values of FA were reported as mean values ± standard deviation (SD) and expressed as relative percentages of the total pool of fatty acids.

### Statistical analysis

Biochemical data were represented by the relative abundance of FA per replicate, per area for each channel. The resemblance matrix among samples was obtained with the Bray-Curtis similarity coefficient, following a log (x + 1) transformation in order to place more emphasis on compositional differences among samples rather than on quantitative differences^22^. A preliminary one-way analysis of similarity (ANOSIM) was performed to detect significant differences in FA profiles of *C*. *edule* between sampling areas within the same channel. Briefly, ANOSIM calculates a global R statistic that assesses the differences between groups, where values close to one indicate maximum differences between groups and values near zero suggest complete groups overlap^23^. As no significant differences were recorded between areas within the same channel, all samples per channel were pooled, i.e. a total of 8 replicates per channel (see Table S2 on supplementary information).

The differences in FA profile in *C. edule* between channels were analysed by ordination analysis, using Principal Coordinates Analysis (PCO). This analysis allows the visualization of inter-individual differences in FA profiles, representing differences between all channels from each FA along the first two axes. ANOSIM was also used (see above for details) to detect differences in FA profiles of the AM of *C. edule* among channels. One-way analysis of variance (ANOVA) was used to assess differences among channels for each individual FA after confirming normality with the Shapiro test and homogeneity of variance with the Bartlett test. Post hoc Bonferroni test was used when ANOVA revealed significant differences (*p* < 0.05).

Similarity percentages (SIMPER) were determined to describe the differences in individual and classes of FA among channels. SIMPER identifies the FAs that contribute most to the variations in the assemblage patterns recorded. Only the FAs that cumulatively contributed up to 80% of the dissimilarities recorded were selected to characterize the differences in the FA profile of cockles from different channels^23^. ANOVAs were performed using GraphPad Prism 6 (GraphPad Software. Inc. San Diego, CA, USA), while all multivariate statistical tests (ANOSIM, PCO and SIMPER) were performed using PRIMER v6 with the add-on PERMANOVA+.

## Results

Saturated FAs (SFA) represented 28–41% of all FAs identified in the AM of *C. edule* from different channels, whereas mono-unsaturated FAs (MUFAs) represented 12–13% (Table 1). Polyunsaturated FAs (PUFAs) were the most abundant class of FAs recorded in the bivalves surveyed as their levels ranged between 45 and 58% of the total pool of FAs. The major SFAs were palmitic (16:0; PA) and stearic acid (18:0), which represented over 50% of all SFAs recorded in the AM of cockles and varied significantly (p < 0.05) among channels (Table 1). The dominant MUFAs were elaidic (18:1*n*-9 trans) and eicosenoic acid (20:1*n*-9), and their content in the AM of cockles was similar across channels (Table 1). The most abundant PUFAs were eicosapentaenoic (EPA) (20:5*n-*3) and docosahexaenoic acid (DHA) (22:6*n-*3), which together accounted for over 60% of total PUFAs and, at least, 35% of the total pool of FAs.

**Figure 2:**
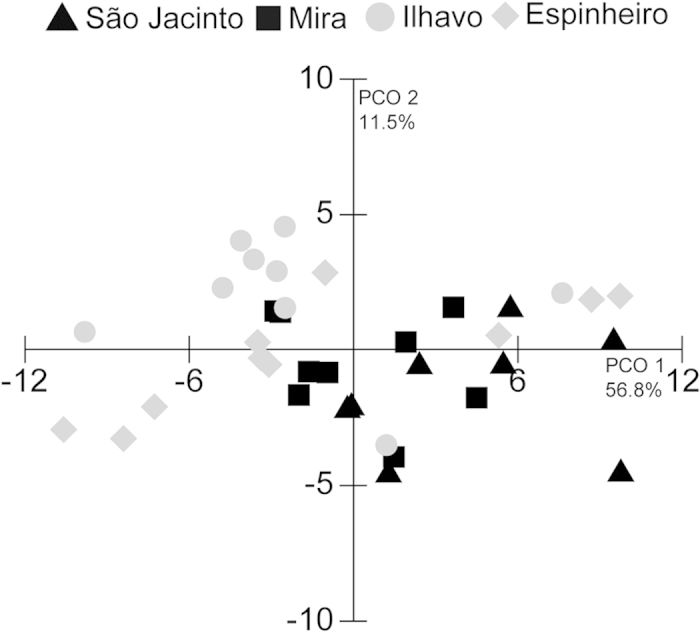
Principal coordinates analysis of the fatty acid composition of the adductor muscle of *Cerastoderma edule* from São Jacinto, Mira, Ilhavo and Espinheiro channels in Ria de Aveiro, Portugal.

The first two axes of the PCO analysis explained 68.3% of the FA variation in the data set (PCO axis 1: 56.8%, PCO axis 2: 11.5%) (Fig. 2). ANOSIM revealed significant differences among FA profiles of *C. edule* from different channels (*p* = 0.041) with the exception of specimens sampled in Ilhavo and Espinheiro (*p* = 0.155). The ANOSIM performed using FA classes (i.e. SFA, MUFA and PUFA) also showed significant differences among channels, apart from São Jacinto and Espinheiro (*p* = 0.059) and Ilhavo and Espinheiro (*p* = 0.296) for SFA, and Mira and Espinheiro Channels (*p* = 0.071) and Ilhavo and Espinheiro Channels (*p* = 0.179) for PUFA (Table 2). SIMPER analysis (Table 3) revealed that PA and DHA were generally among the FAs that most contributed for the differences recorded among channels (e.g. more than 22% of the differences recorded between São Jacinto and Espinheiro were explained by 16:0 and DHA). While specimens originating from Espinheiro and Ilhavo channels showed a relatively similar FA profile, the content of oleic acid (18:1n9c) in the AM of *C. edule* was notably different between these two locations (*p* = 0.0283, Table 1). SIMPER also revealed that myristic acid was responsible for almost 10% of all differences recorded between the pool of FAs displayed by the AM of cockles collected in these two channels (Table 3).

## Discussion

Most of the available studies on the FA profile of bivalves focus on the analysis of their whole body, gonads, gills or digestive gland^24^,^25^,^26^. However, results from the latter two studies reveal notable variations of the FA profile with diet and environmental conditions^24^,^25^. In order to minimize variability of FA profile associated with diet, this study solely analysed the FA content of the AM of *C. edule*^27^. This approach was already successfully employed to discriminate bivalves originating from different locations^16^,^17^ and contrasting habitats (10 m vs. 31 m depth; Napolitano *et al.*^28^). Moreover, Perez *et al.*^26^, combined FA analysis with stable isotopes to assign the location of the origin of bivalves (*Venus verrucosa*) with a spatial resolution <10 Km. The present study shows, for first time, that the FA profile of the AM alone holds to the potential to be used for geographical traceability of bivalves with a similar resolution (<10 Km).

The dominance of PUFAs, followed by SFAs and MUFAs, in the FA profiles of the AM has already been recorded in other bivalve species, such as the fan mussel *Pinna nobilis*^29^, the scallops *Pecten maximus*^16^ and *Placopecten magellanicus*^28^,^30^, in *Astarte sulcata*^17^, the flat oyster *Ostrea edulis*, the black mussel *Mytilus galloprovincialis*, the bearded horse mussel *Modiolus barbatus* and Noah’s ark shell *Arca noae*^31^. In general, and as in the present study, all these works revealed that the dominant SFA was PA, followed by EPA and DHA as the most abundant PUFAs (see Galap *et al.*^32^). PA and DHA were also responsible for most of the differences recorded among Ria de Aveiro channels (Table 1). Both PA and DHA showed significant shifts in their relative abundance with geographical location, which is likely associated with a differential physiological response to variable environmental conditions. Similarities in the FA profile of cockles between São Jacinto and Mira, as well as between Ilhavo and Espinheiro (Table 1; supplementary information), were likely associated with the geographical proximity among these channels and their similar environmental conditions. Despite the major axis of variation (axis 1; Fig. 2) did not clearly separate the specimens originating from each of the four channels of the coastal lagoon, specimens from Ilhavo and Espinheiro channels were mostly separated, which contrasted with specimens from São Jacinto and Mira channels that were relatively spread throughout the PCO. It is worth noting that specimens originating from areas closer to the inlet (São Jacinto and Mira) are likely to be less exposed to lower salinities (Table S1) than those originating from channels located more upstream (Espinheiro and Ilhavo) (Fig. 2). Bivalves further away from the inlet may experience a sharper decrease in salinity during rainfall, due to a notable freshwater contribution of small rivers and streams bordering Ria de Aveiro, and a higher increase in salinity during the summertime promoted by a lower water exchange due to the distance from the inlet and consequent increase in evaporation. Bivalves exposed to higher saline fluctuations are also expected to display a decrease in their levels of SFAs, which is responsible to stabilize the bilayer structure of cell membranes, and an increase in their concentration of PUFAs to enhance bilayer fluidity^24^.

The structural nature of the lipids presents in the AM of bivalves (mostly phospholipids and sterols) provides a stable FA signature that is primarily determined by environmental conditions and functions of the cellular membrane rather than dietary regimes^30^,^33^. At present, most studies available on the FA profiles of bivalves are focused on the analysis of the whole body or other organs than the AM (e.g. gonads, gills and digestive gland)^28^,^34^ that are regulated by intrinsic (e.g. age, phylogeny and sex) and external (e.g. diet, salinity and depth) factors^17^. Conversely, the FA signature of the AM is less prone to fast and dramatic shifts as compared to other organs (e.g. gonads and the digestive gland)^28^,^33^. As this study was carried out in Ria de Aveiro, which is a system with a remarkable spatial variability of environmental conditions among different channels^35^, particularly saline fluctuations, it is likely that environmental variability plays a major role on the FA signature of the AM of cockles from different channels of this ecosystem (see supplementary information).

It is important to note that the FA profile of bivalves may also exhibit temporal variability associated with environmental conditions^31^,^36^. While such temporal variability may be seen as a potential obstacle for using this approach for traceability purposes, it can be circumvented by authorities. For instance, the FA profiles of the AM of control samples collected from the capture/production area claimed as place of origin by the bivalve trader can be matched with those from the batch of bivalves being surveyed. If significant differences are observed, it is likely that the place of origin of the bivalves being traded is not the one claimed by the trader. The average shelf life of fresh cockles traded is generally ≤5 days after harvesting. Therefore, given this limited shelf life of fresh bivalves, it is unlikely to find significant differences in the FA profiles of the AM of control samples and the batch of bivalves being traded caused by the time frame between the collection of both (always ≤5 days). Although the dynamics of the FA profile of cockles during ice storage has never been assessed, a study performed on the whole body of blue mussels *M. edulis* revealed no major shifts on the FA profile during a 14-day storage period in ice^37^. Therefore, while it is likely that the FA profile of *C. edule* may remain stable during a 5-day storage period in ice, this assumption should be tested in the future, specifically for the adductor muscle of *C. edule*.

In conclusion, our null hypothesis that no significant differences in the FA profile of the AM of *C. edule* was expected between specimens captured in different channels of Ria de Aveiro was rejected. Indeed, this biochemical approach allowed us to differentiate cockles originating from different channels of this coastal lagoon. The spatial resolution achieved with this methodological approach revealed the ability to discriminate capture/production areas for bivalves classified as “B” and “C” within the same coastal system (e.g. São Jacinto and Ilhavo). However, this approach was unable to discriminate between areas classified as “C” (Ilhavo and Espinheiro). These findings are important to guarantee that specimens are not mislabelled and illegally traded, as well as address current legislation on seafood traceability^13^. Additionally, geographical traceability may play a key role for fishermen/producers willing to differentiate and add value to their products by assuring that bivalves being traded originate from regions that may be microbiologically safer than others (e.g. displaying lower loads of *Vibrio* spp. or other microorganisms of concern for public health). While this approach is relatively low-cost, it can be further simplified, and performed in a faster way, by employing a direct esterification of the AM followed by extraction^16^,^17^. Future studies should try to apply this methodology to other commercially important bivalves, as well as monitor seasonal and interannual variability to ascertain the suitability of assembling a database for tracing their place of origin.

## Additional Information

**How to cite this article**: Ricardo, F. *et al.* Potential use of fatty acid profiles of the adductor muscle of cockles (*Cerastoderma edule*) for traceability of collection site. *Sci. Rep.* **5**, 11125; doi: 10.1038/srep11125 (2015).

## Supplementary Material

Supplementary Information

## Figures and Tables

**Table 1:** **Fatty acid profile (data presented as percentage of relative abundances) of the adductor muscle of** ***Cerastoderma edule*****(values are means of 8 replicates ± SD) from São Jacinto (SJ), Mira (M), Ilhavo (I) and Espinheiro (E) channels in Ria de Aveiro, Portugal**.

**Table 2:** **Similarity values (ANOSIM) between all fatty acids (FAs), saturated (SFAs) and polyunsaturated (PUFAs) fatty acids in the adductor muscle of** ***Cerastoderma edule*** **from São Jacinto, Mira, Ilhavo and Espinheiro channels in Ria de Aveiro, Portugal.**

**Channels**	**All Fas**		**SFAs**		**PUFAs**		**MUFAs**	
	**R**	***p***	**R**	***p***	**R**	***p***	**R**	***p***
São Jacinto *vs* Mira	0.238	0.020	0.145	0.045	0.222	0.019	0.112	0.056
São Jacinto *vs* Ilhavo	0.358	0.006	0.428	0.002	0.362	0.004	0.046	0.231
São Jacinto *vs* Espinheiro	0.196	0.041	0.135	0.059	0.223	0.023	0.020	0.301
Mira *vs* Ilhavo	0.199	0.017	0.301	0.003	0.147	0.034	−0.010	0.455
Mira *vs* Espinheiro	0.154	0.041	0.182	0.010	0.105	0.071	0.034	0.261
Ilhavo *vs* Espinheiro	0.062	0.155	0.022	0.296	0.050	0.179	−0.009	0.402

**Table 3:** **Similarity percentage analysis (SIMPER) identifying which fatty acids (FAs) contribute to the differences recorded in the adductor muscle of** ***Cerastoderma edule*** **from São Jacinto, Mira, Ilhavo and Espinheiro channels in Ria de Aveiro, Portugal.**
