# Supplementary material for: Potential use of fatty acid profiles of the adductor muscle of cockles (Cerastoderma edule) for traceability of collection site
Source: Sci Rep. 2015 Jun 18;5:11125. doi: 10.1038/srep11125 (PMC4471671; doi:10.1038/srep11125)
Supplement: Supplementary Information [file srep11125-s1.doc]

**Potential use of fatty acid profiles of the adductor muscle of cockles (*Cerastoderma edule*) for traceability of collection site**

**Supplementary information**

Fernando Ricardo^1^, Tânia Pimentel^1^, Ana S.P. Moreira^2^, Felisa Rey^1^, Manuel A. Coimbra^2^, M. Rosário Domingues^2^, Pedro Domingues^2^, Miguel Costa Leal^1^ and Ricardo Calado^1 *^

^1^ Departamento de Biologia & CESAM, Universidade de Aveiro, Campus Universitário de Santiago, 3810-193 Aveiro, Portugal

^2^ QOPNA, Department of Chemistry, University of Aveiro, Campus Santiago, 3810-193 Aveiro, Portugal

Corresponding Author:

*Ricardo Calado

Phone + 351 234 370 779

E-mail: rjcalado@hotmail.com

**Table S1.** Water salinity (S) and linear distances (in km) between the inlet of Ria de Aveiro and sampling locations in São Jacinto (SJ), Mira (M), Ilhavo (I) and Espinheiro (E) channels. Please note that a lower distance to the inlet may not always mean that the location displays a higher water renewal rate or “more marine/less brackish” conditions, as these features are regulated by the geomorphology of the coastal lagoon and consequently its dominant currents.

| **Inlet**  (S 36) |  |  |  |  |  |  |  |  |
| --- | --- | --- | --- | --- | --- | --- | --- | --- |
|  |  |  |  |  |  |  |
| 2.2 | **SJ1**  (S 36) |  |  |  |  |  |  |  |
|  |  |  |  |  |  |  |
| 7.6 | 4.8 | **SJ2**  (S 35) |  |  |  |  |  |  |
|  |  |  |  |  |  |
| 2.8 | 2.1 | 6.9 | **M1**  (S 36) |  |  |  |  |  |
|  |  |  |  |  |
| 9.1 | 8.6 | 13.4 | 6.4 | **M2**  (S 35) |  |  |  |  |
|  |  |  |  |
| 7.9 | 7.2 | 11.9 | 4.8 | 11.5 | **I1**  (S 32) |  |  |  |
|  |  |  |
| 11.6 | 10.2 | 15.0 | 8.3 | 15.0 | 3.2 | **I2**  (S 30) |  |  |
|  |  |
| 6.3 | 5.4 | 10.2 | 3.3 | 10.0 | 3.5 | 6.6 | **E1**  (S 32) |  |
|  |
| 8.4 | 7.8 | 12.5 | 5.5 | 12.1 | 5.7 | 8.9 | 2.4 | **E2**  (S 30) |
|

**Table S2.** Similarity values (ANOSIM) between the FA class profiles of the *Cerastoderma edule* adductor muscle from areas within São Jacinto, Mira, Ilhavo and Espinheiro Channels, Ria de Aveiro, Portugal.

| **Channels** | **R** | ***p*** |
| --- | --- | --- |
|
| São Jacinto 1 vs São Jacinto 2 | 0.271 | 0.114 |
| Mira 1 vs Mira 2 | 0.013 | 0.405 |
| Ilhavo 1 vs Ilhavo 2 | 0.081 | 0.222 |
| Espinheiro 1 vs Espinheiro 2 | 0.116 | 0.190 |
